# Thermal manipulation of the broilers embryos: expression of muscle markers genes and weights of body and internal organs during embryonic and post-hatch days

**DOI:** 10.1186/s12917-019-1917-6

**Published:** 2019-05-23

**Authors:** Mohamed Borhan Al-Zghoul, Sabry Mohamed El-Bahr

**Affiliations:** 1 Department of Basic Medical Veterinary Sciences, Faculty of Veterinary Medicine, Jordan University of Science and Technology, P.O. Box 3030, Irbid, 22110 Jordan; 2 Department of Physiology, Biochemistry and Pharmacology, College of Veterinary Medicine, King Faisal University, P.O. Box 400, Al-Hufof, 31982 Saudi Arabia; 3 Department of Biochemistry, Faculty of Veterinary Medicine, Alexandria University, Alexandria, Egypt

**Keywords:** Muscle, Marker gene, Growth factor, Broiler, Thermal manipulation

## Abstract

**Background:**

In broilers chickens, the molecular bases for promoting muscle development and growth requires further investigation. Therefore, the current study aimed to investigate the effects of daily thermal manipulation (TM) during embryonic days (ED) 12 to 18 on body, carcass and internal organ weights as well as on the expression of muscle growth markers genes during late embryogenesis and post-hatch days. 1500 fertile Cobb eggs were divided into five groups. The first group was a control group and incubated at 37.8°C. The other four groups were thermally manipulated (TM) and exposed to 38.5°C (TM_1_), 39°C (TM_2_), 39.5°C (TM_3_) and 40°C (TM_4_) daily for 18 h, respectively, with a relative humidity of 56%. Body weights (BW) from ED 12 to 18 and on post-hatch days 1, 2, 3, 4, 5, 6, 7, 14, 21, 28 and 35 were recorded. mRNA expression levels of muscle growth factor genes (IGF-1 and GH) and muscle marker genes (Myogenic Differentiation Antigen; MyoD), Myogenin, Pax7, and PCNA) during ED 12 to 18 and on post-hatch days 1, 3, 5, 7, 14 were analyzed. On post-hatch day 35, the carcass and internal organ weights have been also evaluated.

**Results:**

TM during certain days of embryogenesis (ED 12 to 18) did not affect the BW of broilers during their embryonic lives. However, TM, particularly TM_1_ and TM_2_, significantly increased BW, carcass and internal weights of hatched chicks near to the marketing age (post-hatch days 28 and 35). Most of TM protocols induced up-regulation of muscle growth factor genes (IGF-1 and GH) and muscle marker genes (MyoD, Myogenin, Pax7, and PCNA) during embryonic life (ED 12 to 18) and on post-hatch days.

**Conclusion:**

Among the various TM conditions, it seems that,TM_1_ and TM_2_ induced a significant increase in BW, carcass and internal weights of hatched chicks near to the marketing age. This increase in BW induced presumably via up-regulation of muscle growth factor genes and muscle growth markers genes during embryonic life (ED 12 to 18) and on post-hatch days. Both protocols (TM_1_ and TM2) can be used in real-world applications of poultry industry for maximum benefit.

## Background

The rates and efficiencies of muscle tissue deposition vary widely among domestic animals [1, 2]. In muscle cells, as in a variety of cell types, a balance of opposing cellular signals controls proliferation and differentiation [3]. At birth, a high number of proliferating satellite cells are found in skeletal muscle nuclei; however, at the end of the growth phase, their numbers are reduced to less than 5% of total muscle fiber nuclei [4]. The muscle-specific basic helix-loop-helix (bHLH) proteins, a family of transcription factors, regulate the proliferation and differentiation of myoblasts [5]. Upon satellite cell activation, these transcription factors are expressed in a sequential pattern [6]. Initially, Myogenic factor-5 (Myf5) and Myogenic Differentiation Antigen (MyoD) are expressed in the proliferating progeny, followed by myogenin expression as the cells begin to differentiate [7].

Thermal manipulation (TM) has been proposed as a technique to enhance muscle growth and to improve the thermotolerance acquisition of the broiler chicken, in which the embryos are exposed to high or low temperatures during the susceptible period of their lives [8–15]. Previously, it has been shown that the incubation temperature has a significant effect on hatchability rate, chick quality, and post-hatch growth performance parameters [16, 17]. Thermal manipulation (TM) of chicken embryos induced differential gene expression in response to post-hatching heat challenge [18]. In addition, TM immediately affects miRNA expression profiles of breast and hind muscles of chicken embryos and is associated with minor long term expression of miRNAs broiler chickens [19]. However, the molecular bases for promoting muscle development and growth requires further investigation [12, 13, 16, 20]. Therefore, the current study aimed to investigate the effect of different daily TM conditions, namely TM_1_ (38.5°C for 18 h), TM_2_ (39°C for 18 h), TM_3_ (39.5°C for 18 h) and TM_4_ (40°C for 18 h) during embryonic days (ED) 12 to 18, on body (BW) and internal organ weights and expression of muscle growth and transcription factor genes of broiler chickens. The muscle growth factor genes included insulin-like growth factor 1 (IGF-1) and growth hormone (GH), while the muscle marker genes encompassed MyoD, myogenin, paired box transcription factor (Pax7), and proliferating cell nuclear antigen (PCNA) during late embryogenesis and post-hatch days. The results of this study provide an insight into the molecular basis of muscle development and growth of thermally-manipulated (TM) broilers.

## Methods

### Incubation and hatching management

All experimental procedures and management conditions used in this study were approved by Jordan University of Science and Technology’s Animal Care and Use Committee (JUST-ACUC). 1700 fertile Ross broiler eggs were obtained from a certified Ross breeder flock of hens (Irbid, Jordan). The eggs were examined for breakage or any abnormality, with very small (< 59 g) and large eggs (> 68 g) being rejected. 1500 normal eggs with an initial weight of 64 ± 2 g were then selected and incubated in five commercial incubators (Type 25 HS-SSF, Masalles, Barcelona, Spain). The incubators are of continuous turning system, fully digital programmable temperature (room to 70°C) and humidity (20–95%) equipped with fully automated cooling system of self control Natural Cool Down Programmable (NCDP) which avoid temperature swing and equipped with alarm in case of overheating. The temperature inside the incubator was fluctuated with ±0.1°C. The incubator equipped with digital electronic thermostat high precision. The selected eggs were then equally allocated to the following five incubation treatment groups (300 eggs in each group): control group (maintained at 37.8°C and 56% relative humidity (RH) throughout the incubation period); TM_1_ (daily TM at 38.5°C and 65% RH for 18 h on ED 12 to 18); TM_2_ (daily TM at 39°C and 65% RH for 18 h on ED 12 to 18); TM_3_ (daily TM at 39.5°C and 65% RH for 18 h on ED 12 to 18); and TM_4_ (daily TM at 40°C and 65% RH for 18 h on ED 12 to 18). TM1-TM4 were incubated like the control condition (37.8°C; 56% RH) during the last 6 h of the day. Eggs were placed randomly at various locations of the incubators. At hatch, the number of hatched chicks was recorded. In addition, the hatchability (%) was calculated by dividing the number of healthy chicks by the total number of fertilised eggs. One-day old chicks were then transferred to the Animal House at Jordan University of Science and Technology where the experiment was conducted. Chicks were distributed in cages with a starting room temperature of 33°C that was gradually decreased to 24°C on post hatch day 21. From post-hatch day 24 to 35, the temperature was maintained at 21°C. Water and feed were supplied to the chicks ad libitum.

### Sample collection

Pectoral and thigh muscle samples were collected from 100 embryos (10 embryos from each treatment group per day on ED 12 and 18) and from 300 chicks (10 chicks from each treatment group per day on post-hatch days 1, 3, 5, 7, 14 and 28). Euthanasia was performed after sodium pentobarbital anesthesia (20–30 mg/kg; [21]). Sodium pentobarbital was injected to radial vein with sterilized needles. Pectoral muscle mRNA expression levels of muscle marker genes (MyoD, Myogenin, Pax7 and PCNA) and muscle growth factors (IGF-1 and GH) were evaluated using relative quantitative real-time polymerase chain reaction (qRT-PCR) analyses.

At ED 12 and 18, ten embryos from each group were individually weighed using a 0.01 g precision scale. Chicks (*n* = 20) were individually weighed on a daily basis during the post-hatch first week and then on post-hatch days 14, 21, 28 and 35 of age. At post-hatch day 35, 10 birds from each group were euthanized, and the weights of their hearts, lungs, spleens, livers, intestines, and gizzards as well as the lengths of their intestines, caeca, and colons were recorded.

### RNA isolation and qRT-PCR analysis

Total RNA was extracted from homogenated muscles tissues using a TRIzol/choloroform/isopropanol method followed by the removal of supernatants. The RNA pellet was then dissolved in diethylpyrocarbonate treated RNase-free water (Ambion, Texas, USA). DNA was digested using the DNase I kit (Ambion, Austin, TX), and RNA samples were checked for concentration and purity (260:280 nm absorbency). 2 μg RNA was reverse transcribed to create cDNA using the iScript cDNA Synthesis Kit (Bio-Rad, Hercules, CA, USA). qRT-PCR was performed using a QuantiFast SYBR® Green PCR Kit (Qiagen, Valencia, CA, USA) on a Rotor-Gene Q Real-Time PCR system (Qiagen, Valencia, CA, USA). Briefly, the 20 μl reaction mix was prepared from 10 μl of master mix; 2 μl forward primer (10 pmol); 2 μl reverse primer (10 pmol); 2 μl cDNA of the sample and 6.5 μl of nuclease-free water. Cycling parameters were 50°C for 2 min, 95°C for 15 min, 40 cycles of 95°C for 10s, followed by 30s at 55°C and 72°C for 10s with final melting at 95°C for 20s. Post-PCR melting curves confirmed the specificity of single-target amplification, and fold changes in gene expressions were normalized to the housekeeping gene GAPDH. Duplicates from each cDNA was analyzed, fluorescence emission was detected and relative quantification was calculated automatically. The primer sequences that were used in the qRT-PCR analyses are illustrated in Table 1.

**Table 1:** Primers for relative-quantitative real time RT-PCR analyses

Gene	Sequence
cGAPDH	F-5’GTGTTATCATCTCAGCTCCCTCAG’3
cGAPDH	R-5’GGTCATAAGACCCTCCA CAATG3’
cIGF-1	F-5’GTGTTTGCTTACCTTAACCAGTT ‘3
cIGF-1	R-5’GTACAGAGC GTGCAGATT’3
cGH	F-5’ GACCAGAGGTACACCAACAAA3’
cGH	R-5’GACTGGATGAGAACCAGTGAAA3’
cMyoD	F-5’TACCCAGTGCTGGAGCACTA’3
cMyoD	R5’GTCTTGGA GCTTGGCTGAACT’3
cMyogenin	F-5’AGCAGCCTCAACCAGCAGGA’3
cMyogenin	R-5’TCT GCCTGGTCATCGCTCAG’3
cPAX7	F-5’ACTGTGCCCTCAGTGAGTTCGATT’3
cPAX7	R-5’ATTCGACATCGGAGCCTTCATCCA ‘3
cPCNA	F-5’ACGCATTTGTAGAGACC TCAGCCA
cPCNA	R-5’AGTCAGCTGGACTGGCTCATTCAT’3

GAPDH, glyceraldehyde 3-phosphate dehydrogenase; IGF-1, insulin-like growth factor 1; GH, growth hormone; MyoD, myogenic differentiation gene; Pax7, paired box transcription factor; PCNA, proliferating cell nuclear antigen

### Statistical analysis

All statistical analyses were performed using IBM SPSS statistics 23 software (IBM software, Chicago, USA). Hatchability was analyzed by the Chi-square test. Data for BW, internal organ weights, muscle marker and growth factor gene expression were conveyed in means ± SD. One-way ANOVA followed by the all-pairs Bonferroni test was used to compare different parameters in all treatment groups, and differences were considered significant at *P* < 0.05.

## Results

### Effect of thermal manipulation on hatchability and embryonic and post-hatch body weight

Eggs from which embryos taken for measurement of embryonic mRNA levels of muscle growth markers genes and were excluded from the hatchability results. The effect of TM during embryogenesis on hatchability is summarized in Table 2. The total number of hatched eggs after 21 days of incubation was 1045. TM1 and TM2 did not affect the hatchability percentage and remained comparable to that of the control. However, TM3 and TM4 reduced the hatchability percentage compare to control, TM1 and TM2.

**Table 2:** The effect of thermal manipulation (TM) on hatchability in broiler chicks subjected to different thermal manipulation treatments during embryogenesis

Parameters	Groups				
	Control	TM_1_	TM_2_	TM_3_	TM_4_
Non-hatched eggs	38 (16.24%)	41 (16.27%)	42 (16.67%)	62 (22.46%)	64 (23.03%)
Total (hatch)	196/234	211/252	210/252	214/276	214/278
Hatchability %	83.76^a^	83.73^a^	83.33^a^	77.53^b^	76.97 ^b^

^a-b^ Within the same ED, means ± SD with different superscripts differ significantly (*P* < 0.05)

**Fig. 1:**
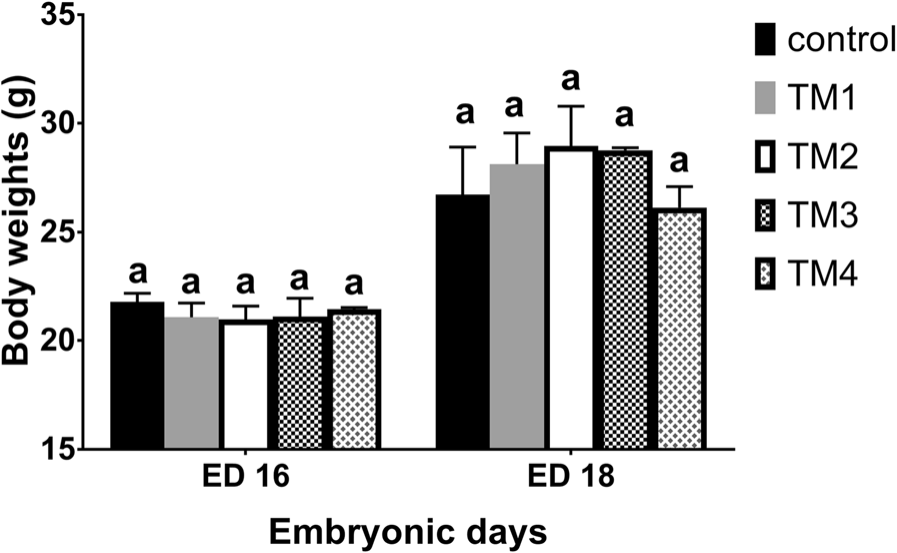
The effect of thermal manipulation (TM) on embryonic body weight in broiler chicks subjected to different thermal manipulation treatments during embryogenesis) *n* = 3). Control = 37.8°C for 24 h; TM_1_ = Thermal manipulation at 38.5°C for 18 h; TM_2_ = Thermal manipulation at 39°C for 18 h; TM_3_ = Thermal manipulation at 39.5°Cfor 18 h, TM_4_ = Thermal manipulation at 40°C for 18 h. ^a-b^ Within the same ED, means ± SD with different superscripts differ significantly (*P* < 0.05)

**Fig. 2:**
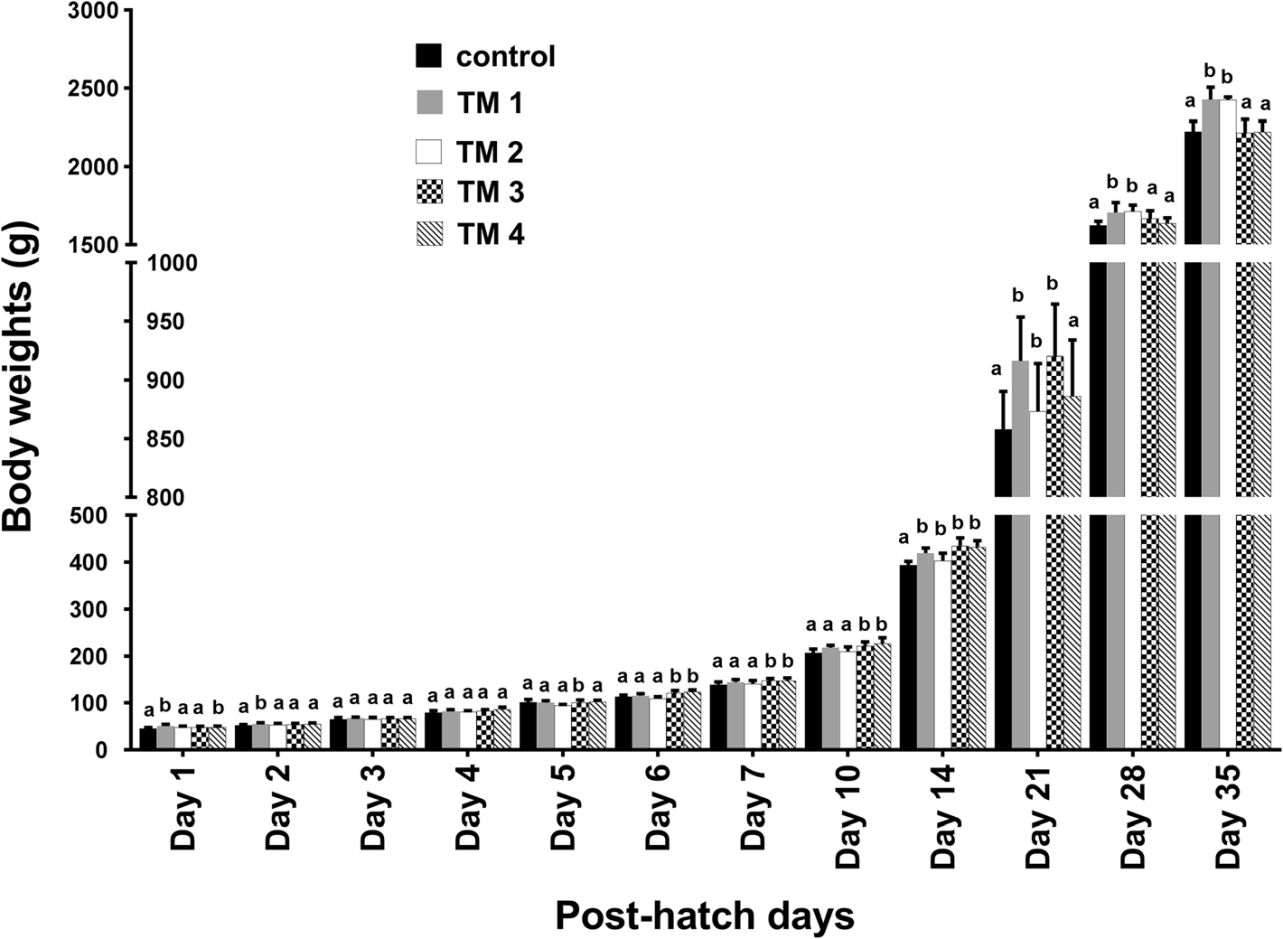
The effect of thermal manipulation (TM) during embryogenesis on body weight in post-hatch broiler chicks from day 1 to day 35 (*n* = 20). Control = 37.8°C for 24 h; TM_1_ = Thermal manipulation at 38.5°C for 18 h; TM_2_ = Thermal manipulation at 39°C for 18 h; TM_3_ = Thermal manipulation at 39.5°Cfor 18 h, TM_4_ = Thermal manipulation at 40°C for 18 h. ^a–b^ Within the same day, means ± SD with different superscripts differ significantly (*P* < 0.05)

The effect of TM on embryonic BW is shown in Fig. 1. Similar embryonic BW were observed for both the treatment and control groups. The BW of chicks in the post-hatch period (first week of age and post-hatch day 10, 14, 21, 28 and 35) is shown in Fig. 2. TM chicks exhibited an advantage over controls up to 35 day of age with regard to BW. On post-hatch day 1, BW of chicks in the TM_1_ and TM_4_ groups were significantly (*p* < 0.05) higher than those in the TM_2_ and TM_3_ groups, both of which remained comparable to controls. On post-hatch day 2 and 3, the BW of all treatments were comparable to the controls (*p* > 0.05). On post-hatch day 4, BW of the TM_4_ group was significantly (*p* < 0.05) higher than that of the other treatment groups which remained comparable to the control group. On post-hatch day 5, BW of the TM_3_ group was significantly (*p* < 0.05) higher than that of other treatment groups which remained comparable to the controls. On post-hatch days 6, 7, and 10, BW of TM_3_ and TM_4_ groups were significantly (*p* < 0.05) higher than that of the TM_1_ and TM_2_ groups. On post-hatch day 21, the BW of the TM_1_ and TM_3_ groups was significantly (*p* < 0.05) higher when compared with the other treatment groups. Interestingly, on post-hatch day 28 and 35, BW of the TM_1_ and TM_2_ groups was significantly (p < 0.05) higher than that of other TM groups (Fig. 2 and Table 3).

**Table 3:** Body weight, carcass weight, breast muscle weights and weight/or length of visceral organs at day 35 of age in broiler chicks subjected to different thermal manipulation protocols during embryonic life (ED12–18)

Parameters	Groups				
	Control	TM_1_	TM_2_	TM_3_	TM_4_
Body weight	2222.98 ± 65.68^a^	2426.64 ± 70.39^b^	2425.78 ± 17.29^b^	2216.22 ± 85.39^a^	2220.37 ± 69.66^a^
Carcass weight	1423.90 ± 69.46^a^	1568.87 ± 24.83^b^	1569.27 ± 16.36^b^	1521.52 ± 06.69^c^	1515.92 ± 06.75^c^
Breast muscle weight	493.05 ± 28.98^a^	571.07 ± 08.45^b^	563.02 ± 20.10^b^	580.54 ± 54.39^b^	584.75 ± 40.09^b^
Breast muscle weight/ Carcass weight %	34.62 ± 00.44^a^	37.12 ± 01.04^b^	36.81 ± 00.81^b^	37.62 ± 00.90^b^	38.55 ± 01.02^b^
Heart weight	13.41 ± 00.87^a^	15.57 ± 00.80^b^	15.54 ± 00.70^b^	14.68 ± 01.62^a^	14.53 ± 01.76^a^
Lung weight	13.46 ± 00.01	12.58 ± 00.13	13.08 ± 00.50	13.24 ± 01.06	12.30 ± 02.12
Spleen weight	2.26 ± 00.25	2.45 ± 00.45	2.44 ± 00.10	2.33 ± 00.94	01.92 ± 00.47
Liver weight	47.15 ± 02.25^a^	56.00 ± 03.47^b^	54.57 ± 02.71^b^	52.60 ± 06.73^b^	51.52 ± 02.26^b^
Intestine weight	127.11 ± 06.93	130.40 ± 10.85	132.22 ± 05.06	131.82 ± 00.69	124.27 ± 06.09
Gizzard weight	47.69 ± 03.03^a^	45.51 ± 00.13^a^	42.50 ± 01.87^a^	59.55 ± 06.61^b^	46.84 ± 01.92^a^
Intestine length	167.33 ± 02.31	171.33 ± 04.73	164.67 ± 01.53	165.50 ± 03.54	165.67 ± 04.40
Cecum length	17.00 ± 01.00	16.57 ± 00.58	17.00 ± 01.00	19.33 ± 01.15	17.03 ± 01.00
Colon length	20.33 ± 02.02^a^	16.67 ± 00.58^b^	16.00 ± 01.00^b^	18.67 ± 01.53^a^	16.67 ± 00.58^b^

TM: Thermal manipulation

^a-c^ Within the same row, means ± SD with different superscripts differ significantly (*P* < 0.05)

### Effect of thermal manipulation on post-hatch internal organs weights

On post-hatch day 35, TM induced a significant (*p* < 0.05) increase in carcass weight, breast muscle weight, breast weight/carcass weight percentage and liver weight compared to control (Table 3). On post-hatch day 35, carcass weight of the TM_1_ and TM_2_ groups was significantly (p < 0.05) higher than that of other TM groups (Table 3). Interterestingly, on post-hatch day 35, the heart weight and colon length of TM_1_ and TM_2_ were higher (*p* < 0.05) than whose of other treatment and control groups (Table 3). However, on post-hatch 35, lung, spleen, and intestine weights as well as intestine and cecum lengths were not changed (*p* > 0.05) in all TM groups compared to controls (Table 3). Finally, on post-hatch day 35, gizzard weights of those in the TM_3_ group was higher (*p* < 0.05) than that of the other TM and control groups (Table 3).

### Effects of thermal manipulation on mRNA expression levels of muscle growth factors (IGF-1 and GH) and muscle marker genes (MyoD, Myogenin, Pax7 and PCNA) on ED 12 and 18

The effects of TM on mRNA expression levels of muscle growth factors and marker genes on ED 12 and 18 are illustrated in Table 4. On ED 12, TM induced significant elevation of MyoD levels except for TM_3_, which remained comparable to controls. Furthermore, TM induced significant (p < 0.05) elevation to myogenin level compared to control except for TM_3_ which reduced the expression of this gene. Furtherly, TM_3_ and TM_4_ significantly induced IGF-1 upregulation, while TM_1_ and TM_2_ downregulated the expression of this gene compared to controls. In addition, TM_1_, TM_2_ and TM_3_ increased the GH level compared to TM_4_ which remained comparable to the control (Table 4). TM_2_ induced overexpression of this gene followed by TM_1_ and TM_3_ (Table 4). TM_4_ increased PAX7 levels compared to other TM groups, which reduced its levels significantly compared to controls. Finally, TM_2_ and TM_4_ increased PCNA levels compared to those of TM_1_ and TM_3_.

**Table 4:** Relative mRNA levels of MyoD, Myogenin, IFG-1, GH, PAX7 and PCNA in the muscle tissues on embryonic days (ED) 12 and 18 in broiler chicks subjected to different thermal manipulation protocols during embryonic life (ED12–18)

ED	Parametrs	Groups				
		Control	TM_1_	TM_2_	TM_3_	TM_4_
ED12	MyoD	1 ± 0.01^a^	1.11 ± 0.02^b^	1.14 ± 0.03^b^	1.02 ± 0.03^a^	1.63 ± 0.07^c^
	Myogenin	1 ± 0.01^a^	1.38 ± 0.03^b^	1.68 ± 0.06^c^	0.94 ± 0.02^d^	1.16 ± 0.04^e^
	IGF-1	1 ± 0.01^a^	0.86 ± 0.02^b^	0.92 ± 0.03^b^	1.18 ± 0.01^c^	1.55 ± 0.08^d^
	GH	1 ± 0.05^a^	8.90 ± 0.04^b^	15.8 ± 0.05^c^	1.47 ± 0.04^d^	1.03 ± 0.05^a^
	PAX7	1 ± 0.01^a^	0.85 ± 0.02^b^	0.92 ± 0.04^b^	0.79 ± 0.02^b^	1.38 ± 0.04^c^
	PCNA	1 ± 0.01^a^	1.07 ± 0.02^a^	1.11 ± 0.01^b^	0.99 ± 0.02^a^	1.19 ± 0.03^b^
ED18	MyoD	1 ± 0.02^a^	1.59 ± 0.02^b^	1.85 ± 0.03^b^	1.88 ± 0.03^b^	2.31 ± 0.07^c^
	Myogenin	1 ± 0.11^a^	2.57 ± 0.53^b^	4.05 ± 0.16^c^	2.79 ± 0.12^b^	21.9 ± 0.44^d^
	IGF-1	1 ± 0.01^a^	1.29 ± 0.02^b^	1.72 ± 0.03^c^	1.37 ± 0.01^b^	1.85 ± 0.05^d^
	GH	1 ± 0.05^a^	4.11 ± 0.04^b^	18.60 ± 0.05^c^	4.63 ± 0.04^b^	5.33 ± 0.25^d^
	PAX7	1 ± 0.03^a^	1.43 ± 0.02^b^	1.21 ± 0.04^c^	1.40 ± 0.02^b^	2.18 ± 0.07^d^
	PCNA	1 ± 0.06^a^	2.93 ± 0.02^b^	3.96 ± 0.19^c^	2.12 ± 0.10^b^	2.50 ± 0.10^b^

TM: Thermal manipulation; ED: embryonic day; MyoD: Myogenic differentiation gene; IGF-1: Insulin-like growth factor 1; GH: Growth hormone; Pax7: Paired box transcription factor; PCNA: Proliferating cell nuclear antigen

^a-e^ Within the same row, means ± SD with different superscripts differ significantly (*P* < 0.05)

On ED 18, TM induced significant increases in MyoD levels compared to controls, but this effect was more pronounced in the TM_4_ group. TM induced a significant elevation in myogenin expression compared to controls, however, this effect was more pronounced in the TM_4_ group. Additionally, TM induced significant overexpression in IGF-1 and GH levels compared to controls, and TM_2_ induced overexpression of GH compared to other TM groups. Finally, TM induced significant elevation of the PAX7 and PCNA genes compared to controls.

### Effects of thermal manipulation on mRNA expression levels of muscle growth factors (IGF-1 and GH) and muscle marker genes (MyoD, Myogenin, Pax7 and PCNA) on post-hatch days 1, 3, 5 and 7

On post-hatch 1, TM, except for TM_2_, induced significant elevation in MyoD levels, and the highest effect was reported in TM_3_. In contrast, myogenin expression was downregulated by TM, except for TM_1_ which remained comparable to the controls, and the lowest myogenin expression was observed in TM_2_. TM induced significant reduction in IGF-1 levels compared to control, whereas no significant changes were documented in the GH levels of all TM groups on post-hatch days 1 and 3. Moreover, TM induced a significant increase in PAX7 levels compared to the control group on post-hatch days 1, 3, 5. 14, and 28. TM significantly decreased PCNA levels compared to controls.

On post-hatch day 3, TM_1_ induced significant increases in MyoD and myogenin levels compared to other TM groups. TM_1_ and TM_4_ significantly elevated IGF-1 and PCNA levels, whereas TM_2_ and TM_3_ significantly reduced IGF-1 levels compared to controls.

On post-hatch day 5, TM_3_ and TM_4_ significantly reduced MyoD levels. TM, except for TM_1_, upregulated myogenin expression, with TM_4_ resulting in the highest myogenin expression. TM, except for TM_1_, significantly reduced IGF-1 levels. TM induced upregulation of GH expression, with the highest GH expression being observed in the TM_4_ and TM_3_ groups. Moreover, TM induced significant PCNA elevation compared to the controls.

On post-hatch day 7, TM_4_ induced significant elevation in MyoD levels. TM_1_ and TM_4_ caused an elevation in myogenin expression, while TM_3_ significantly reduced it. TM increased IGF-1 levels but significantly reduced its levels. GH expression was significantly elevated by TM (except for TM_3_). No significant changes in the level of PAX7 expression have been detected in all treatment groups compared to controls. In addition, TM_1_, TM_2_ and TM_4_ increased the PCNA level compared to TM_3_ which remained comparable to the control (Table 5). The expression of this gene was more pronnunceed in tissues of TM_4_ compare to TM_1_ and TM_2_ (Table 5).

**Table 5:** Relative mRNA levels of MyoD, Myogenin, IFG-1, GH, PAX7 and PCNA in the muscle tissues at days 1, 3, 5, 7 post hatch in broiler chicks subjected to different thermal manipulation protocols during embryonic life (ED12–18)

Post-Hatch days	Parameters	Groups				
		Control	TM_1_	TM_2_	TM_3_	TM_4_
Day 1	MyoD	1 ± 0.01^a^	1.80 ± 0.03^b^	1.13 ± 0.02^a^	3.46 ± 0.06^c^	1.25 ± 0.02^d^
	Myogenin	1 ± 0.01^a^	0.89 ± 0.02^a^	0.25 ± 0.02^b^	0.71 ± 0.02^c^	0.69 ± 0.02^c^
	IGF-1	1 ± 0.01^a^	0.63 ± 0.02^b^	0.44 ± 0.03^b^	0.39 ± 0.01^b^	0.62 ± 0.03^b^
	GH	1 ± 0.02^a^	1.26 ± 0.01^b^	1.15 ± 0.01^c^	1.15 ± 0.04^c^	1.09 ± 0.01^c^
	PAX7	1 ± 0.01^a^	1.82 ± 0.01^b^	1.40 ± 0.06^c^	1.91 ± 0.07^b^	1.21 ± 0.07^c^
	PCNA	1 ± 0.01^a^	0.65 ± 0.02^b^	0.55 ± 0.02^b^	0.40 ± 0.02^b^	0.31 ± 0.02^b^
Day 3	MyoD	1 ± 0.01^a^	2.46 ± 0.03^b^	0.82 ± 0.02^a^	0.65 ± 0.02^a^	1.22 ± 0.02^a^
	Myogenin	1 ± 0.01^a^	2.05 ± 0.03^b^	1.28 ± 0.03^a^	0.59 ± 0.02^a^	1.46 ± 0.01^a^
	IGF-1	1 ± 0.01^a^	2.47 ± 0.06^b^	0.63 ± 0.03^d^	0.65 ± 0.03^d^	1.56 ± 0.03^c^
	GH	1 ± 0.02^a^	1.73 ± 0.06^b^	1.01 ± 0.01^a^	0.04 ± 0.04^c^	1.55 ± 0.01^b^
	PAX7	1 ± 0.02^a^	3.29 ± 0.04^b^	1.55 ± 0.01^c^	1.46 ± 0.02^c^	1.99 ± 0.02^c^
	PCNA	1 ± 0.03^a^	3.18 ± 0.09^b^	1.05 ± 0.01^a^	1.03 ± 0.02^a^	1.63 ± 0.05^c^
Day 5	MyoD	1 ± 0.02^a^	0.95 ± 0.03^a^	0.79 ± 0.03^a^	0.35 ± 0.02^b^	0.13 ± 0.01^b^
	Myogenin	1 ± 0.06^a^	1.45 ± 0.02^a^	3.49 ± 0.23^b^	4.36 ± 0.19^c^	10.54 ± 0.27^d^
	IGF-1	1 ± 0.02 ^a^	1.33 ± 0.05^a^	0.64 ± 0.03^a^	0.34 ± 0.01^b^	0.15 ± 0.01^b^
	GH	1 ± 0.03^a^	1.37 ± 0.06^a^	6.77 ± 0.02^b^	7.27 ± 0.02^c^	8.50 ± 0.07^d^
	PAX7	1 ± 0.03^a^	1.11 ± 0.04^b^	1.51 ± 0.02^b^	1.17 ± 0.01^b^	1.15 ± 0.03^b^
	PCNA	1 ± 0.09^a^	1.33 ± 0.04^a^	1.31 ± 0.02^a^	1.69 ± 0.06^b^	5.01 ± 0.13^c^
Day 7	MyoD	1 ± 0.04^a^	1.46 ± 0.11^a^	1.10 ± 0.03^a^	1.12 ± 0.04^a^	2.09 ± 0.06^b^
	Myogenin	1 ± 0.01^a^	2.04 ± 0.22^b^	1.28 ± 0.05^a^	0.05 ± 0.19^d^	4.63 ± 0.19^c^
	IGF-1	1 ± 0.07^a^	1.54 ± 0.11^b^	1.70 ± 0.05^b^	1.65 ± 0.03^b^	0.14 ± 0.02^c^
	GH	1 ± 0.01^a^	2.44 ± 0.15^b^	1.37 ± 0.07^a^	1.02 ± 0.20^a^	5.80 ± 0.10^d^
	PAX7	1 ± 0.03	1.01 ± 0.06	1.18 ± 0.05	1.01 ± 0.01	1.01 ± 0.03
	PCNA	1 ± 0.03^a^	1.65 ± 0.06^b^	1.28 ± 0.05^b^	1.01 ± 0.06^a^	2.56 ± 0.20^c^

TM: Thermal manipulation; MyoD: Myogenic differentiation gene; IGF-1: Insulin-like growth factor 1; GH: Growth hormone; Pax7: Paired box transcription factor; PCNA: Proliferating cell nuclear antigen. ^a-d^ Within the same row, means ± SD with different superscripts differ significantly (*P* < 0.05)

### Effects of thermal manipulation on mRNA expression levels of muscle growth factors (IGF-1 and GH) and muscle marker genes (MyoD, Myogenin, Pax7 and PCNA) on post-hatch days 14 and 28

On post-hatch day 14, TM, except for TM_1_, induced significant elevation in MyoD levels, with the most pronounced expression being in TM_3_. TM_1_, TM_2_ and TM_4_ reduced myogenin and GH levels, while TM_3_ significantly elevated it compared to controls (Table 6). TM upregulated IGF-1 and PCNA gene expression compared to controls (Table 6). The expressions of IGF-1 gene was more pronnunceed in tissues of TM_3_ followed by TM_2_ > TM_4_ > TM_1_ (Table 6). TM_2_, TM_3_ and TM_4_ elevated PAX7 level compare to TM_1_ which remained comparable to control (Table 6). On post-hatch day 28, TM induced significant elevation of MyoD levels compared to controls, and this effect was most noticeable in the TM_2_ group (Table 6). TM_2_ and TM_4_ significantly upregulated myogenin expression while TM_1_ downregulated myogenin expression compared to other TM_3_ and control (Table 6). TM upregulated the expression of IGF-1, GH, PAX7 and PCNA genes compared to controls (Table 6). Compared to control, the patterns of increase in expression of IGF-1, GH, PAX7 and PCNA genes followed these orders, respectively TM_2_ > TM_3_ > TM_4_ > TM_1_; TM_2_ > TM_3_ = TM_4_ > TM_1_; TM_2_ > TM_4_ > TM_1_ > TM_3_; TM_2_ = TM_4_ > TM_1_ = TM_3_ (Table 6).

**Table 6:** Relative mRNA levels of MyoD, Myogenin, IFG-1, GH, PAX7 and PCNA in the muscle tissues at days 14 and 28 post hatch in broiler chicks subjected to different thermal manipulation protocols during embryonic life (ED12–18)

Post-Hatch days	Parameters	Groups				
		Control	TM_1_	TM_2_	TM_3_	TM_4_
Day 14	MyoD	1 ± 0.03^a^	1.16 ± 0.06^a^	1.53 ± 0.03^b^	2.29 ± 0.01^c^	1.64 ± 0.07^b^
	Myogenin	1 ± 0.06^a^	0.44 ± 0.02^b^	0.79 ± 0.03^c^	1.67 ± 0.06^d^	0.49 ± 0.01^b^
	IGF-1	1 ± 0.04^a^	1.40 ± 0.04^b^	1.92 ± 0.05^c^	2.25 ± 0.02^d^	1.60 ± 0.09^e^
	GH	1 ± 0.01^a^	0.39 ± 0.01^b^	0.56 ± 0.07^b^	1.40 ± 0.06^c^	0.51 ± 0.01^b^
	PAX7	1 ± 0.03^a^	1.09 ± 0.06^a^	1.34 ± 0.05^b^	2.02 ± 0.01^c^	1.29 ± 0.03^b^
	PCNA	1 ± 0.03^a^	1.28 ± 0.05^b^	1.63 ± 0.03^b^	1.56 ± 0.06^b^	1.73 ± 0.04^b^
Day 28	MyoD	1 ± 0.03^a^	1.55 ± 0.03^b^	3.44 ± 0.11^c^	1.53 ± 0.02^b^	1.78 ± 0.01^b^
	Myogenin	1 ± 0.06^a^	0.10 ± 0.22^b^	4.44 ± 0.05^c^	1.04 ± 0.08^a^	1.30 ± 0.02^d^
	IGF-1	1 ± 0.04^a^	1.48 ± 0.07^b^	3.53 ± 0.05^c^	2.99 ± 0.05^d^	2.53 ± 0.07^e^
	GH	1 ± 0.01^a^	1.12 ± 0.01^b^	4.09 ± 0.07^d^	1.93 ± 0.05^c^	1.17 ± 0.06^c^
	PAX7	1 ± 0.03^a^	1.96 ± 0.06^b^	3.44 ± 0.13^c^	1.53 ± 0.06^d^	2.21 ± 0.13^b^
	PCNA	1 ± 0.03^a^	1.15 ± 0.02^b^	1.89 ± 0.16^c^	1.19 ± 0.03^b^	1.63 ± 0.05^c^

TM: Thermal manipulation; MyoD: Myogenic differentiation gene; IGF-1: Insulin-like growth factor 1; GH: Growth hormone; Pax7: Paired box transcription factor; PCNA: Proliferating cell nuclear antigen

^a-e^ Within the same row, means ± SD with different superscripts differ significantly (*P* < 0.05)

## Discussion

Previously, TM during mid- or late-term broiler embryogenesis has been shown to enhance muscle hypertrophy due to the increasing proliferation and differentiation of both fetal and adult myoblasts, leading to an increased myogenic cell pool in the embryo and posthatch broilers [15, 20]. In the current study TM1 (38.5°C) and TM2 (39°C) for 18 h did not affect the hatchability percentage while TM3 (39.5°C) and TM4 (40°C) for 18 h reduced the hatcahbiltiy perecentage significantly compre to other groups incuding the control. Previous studies by others have reported that TM (40°C) for 24 h at ED16–18 [22] and TM (39.5°C) for 12 or 24 h at ED7–16 [10, 11] resulted in the improvement of thermotolerance acquisition, with a significant adverse effect on hatchability of broiler chickens. While TM (39.5°C) for 3, 12 or 24 h at ED16–18 had not only improved thermotolerance acquisition, but also resulted in an enhanced hatchability [9].

In this study, TM did not affect the embryonic weights of broiler chicken during ED 12 and 18. This finding agrees with previous report [23] using other themal manipulation protocols (39.6°C at 60% relative humidity for 6 h daily from 0 to 8th day, and 39.6°C at 60% relative humidity for 6 h daily from the 10 to 18th day) in broiler chickens. Earlier report [24] demonstrated thatTM (39.5°C and 65% RH for 12 h/d from ED7 to ED16) did not affect the male BW but females BW were lower than that of male during the entire 70 day posthatching study in broilers chicken.

However, BW and internal organ weights of hatched chicks were increased significantly as a result of TM, particularly chicks in the TM_1_ and TM_2_ groups near to the marketing age (post-hatch days 28 and 35). Conversely, embryos subjected to TM (39.5°C during ED 12–18 for 6 h daily), during satellite cell population expansion experienced increased myofiber diameters and absolute muscle growth in treated chicks relative to controls until post-hatch day 35 [20]. In addition, it has been reported that mild heat exposure at an early age results in the acceleration of satellite cell myogenesis mediated by specific local growth factor expression [25]. This confliction can be attributed to the incubation period differences in the current study contrasted with other reports. The significant increase in weights of liver and heart tissues as a result of TM in the current study disagrees with previours reports in ostrrish [26] and Egyptian chickens [27] exposed to high incubation temperature during their late embryonic life. The significant increase in the breast muscles of birds exposed to TM of the current study are in the line with that obesreved earlier [10, 28] in the broiles chickens.

Myoblast proliferation and differentiation processes are controlled by the muscle-specific basic helix-loopost-hatch elix (bHLH) family of transcription factors [5]. The MyoD family contains four basic helix-loop-helix (bHLH) transcription factors (MyoD, myogenic factor-5 (Myf5), myogenin, and myogenic regulatory factor-4 (MRF4) and positively regulates myogenesis [5]. These factors are expressed in a sequential pattern when satellite cells are activated [5]. Initially, Myf5 and MyoD are expressed in the proliferating progeny, after which myogenin is expressed as the cells begin to differentiate [29]. The current findings showed that TM resulted in immediate stimulation of muscular MyoD mRNA expression in all TM groups on ED 12 and 18, with the highest expression being detected in TM_4_. At post-hatch days 14, and 28, a significant increase in MyoD expression was observed in all TM groups. It seems that TM has early- (ED 12 and 18) and late-term (post-hatch days 7, 14 and 28) effects in myoblast proliferation as indicated by the increase of MyoD mRNA expression. In this study, TM resulted in immediate stimulation of muscular myogenin mRNA expression on ED 18 in all TM groups. On post-hatch day 28, a higher expression of myogenin was only observed in the TM_2_ and TM_3_ groups. Similarly, it has been reported that TM (39.5°C at ED 12–18 for 3 and 6 h) resulted in the enhancement of cell differentiation as indicated by muscular myogenin expression levels [20]. The current findings indicate that TM resulted in an immediate stimulation (early-term effect) of myogenin mRNA expression during embryogenesis and a delayed stimulation on post-hatch days 7, 14 and 28 (late-term effect). These results are in contrast with the previous findings [20] which reported that the increase of muscle hypertrophy in heat-treated chicks was similar,regardless of TM length (3 h vs 6 h).

Pax7 has been suggested as an early marker of myogenesis during post-hatch muscle growth, and its expression is sustained by satellite cells in adult chicken muscle [30, 31]. Pax7 plays a key role in the formation of adult mouse skeletal muscle [32]. Furthermore, Pax7 is expressed during myoblast proliferation and decreases during differentiation [30]. The results of the current study illustrated that TM resulted in immediate stimulation of muscular Pax7 mRNA expression on ED 12 and 18, with a higher expression of Pax7 detected in TM_4_. Furthermore, Pax7 expression significantly increased on post-hatch days 1, 3, 5, 14, and 28.

Previous study [20] reported that, TM (39.5°C at ED 12–18) resulted in immediate (ED17) and later (up to 2 weeks post-hatch) effects in myoblast proliferation as indicated by higher DNA incorporation of thymidine, higher number of muscle cells expressing PCNA in intact muscle, and higher Pax7 protein levels. The aforementioned study suggested that TM enhanced muscle hypertrophy by stimulation of the myogenic progeny cell (satellite cell) proliferation. In this study, TM_2_ and TM_4_ resulted in a significant increase of muscular PCNA mRNA expression, and the highest PCNA expression was detected on ED 12 and 18 as well as on post-hatch days 7, 14, and 28.

In mammals and birds, muscle cell proliferation and differentiation are controlled by growth factors and hormones that play a pivotal role in promoting the growth of skeletal muscle [33]. One of the most important growth factors that regulates the proliferation of satellite cells is insulin growth factor-1 (IGF-1), the latter of which plays a role in both muscle growth and hypertrophy via its effect on satellite cells [33, 34]. Previously, a higher level of IGF-1 gene expression was observed in chicken breast muscle tissues in response to early age TM (24 h on post-htach day 3) [25], TM at 38.5°C on ED 12 to 18 [35], and TM at 39.5°C on ED 12 to 18 [20]. In this study, TM resulted in the immediate stimulation of muscular IGF-1 mRNA expression during ED 18 and on post-hatch days 5, 7, 14, and 28. This increase of IGF-1 expression was associated with significant increases in BW of thermally manipulated chicks on post-hatch days 28 and 35.

Growth hormone plays a pivotal role in promoting the growth of mammalian skeletal muscle, and it has been shown that administration of GH to humans, ruminants, and pigs enhanced their skeletal muscle growth [36–41]. Interestingly, IGF-I has been suggested to mediate the effect of GH on muscle growth, as IGF-I mRNA levels are also stimulated in liver tissue and skeletal muscle as a result of GH administration [38]. The current data indicate that TM resulted in immediate stimulation of muscular GH mRNA expression on ED 12 and 18 and on post-hatch days 7 and 14 in all TM groups, while, on post-hatch day 28, the only significant increase observed is in the TM_3_ group. However, the regulation of IGF-1 and muscle tissues growth by GH of pituitary origin can not be ignored.

## Conclusion

Together, these findings suggest a differential effect of various TM periods on muscle growth and development and it appears that TM_1_ and TM_2_ presented the optimum conditions for its enhancement. This was evident by the significant increases in embryonic and post-hatch BW on days 28 and 35. This improvement in BW was associated with immediate and long-lasting effects of TM in the expression of muscle growth factors (IGF-1 and GH) and muscle marker genes (MyoD, Myogenin, Pax7, and PCNA). The current study concluded that TM improved the BW and up-regulated the muscle growth factor and marker genes. Moreover, it seems that TM_1_ and TM_2_ were superior protocols that can be used to attain the objectives of the current study.

## Data Availability

The datasets used and/or analyzed during the current study are available from the corresponding author on reasonable request.

## Abbreviations

ANOVA: Analysis of variance
bHLH: Basic helix-loop-helix
BW: Body weight
ED: Embryonic days
GH: Growth hormone
IGF-1: Insulin-like growth factor 1
MRF4: Myogenic regulatory factor-4
Myf5: Myogenic factor-5
MyoD: Myogenic differentiation gene
Pax7: Paired box transcription factor
PCNA: Proliferating cell nuclear antigen
RH: Relative humidity
TM: Thermal manipulation
